# The Evolving Field of Tumor Immunopharmacology

**DOI:** 10.3390/ph18101570

**Published:** 2025-10-18

**Authors:** Marco A. Velasco-Velázquez

**Affiliations:** School of Medicine, Universidad Nacional Autónoma de México (UNAM), Mexico City 04510, Mexico; marcovelasco@unam.mx

Tumor immunopharmacology has evolved from the use of broad, highly toxic immune stimulants to the development of targeted immunomodulators [1]. Today, cancer treatment includes monoclonal antibodies directed against immune checkpoints or specific tumor markers, adoptive cell therapies, therapeutic cancer vaccines, and other immunomodulators. For instance, since 2014, the U.S. FDA has approved ten therapeutic antibodies targeting the PD-1/PD-L1 axis for use in multiple solid tumors and lymphomas [2]. Immunotherapy has thus become one of the pillars of modern cancer treatment.

The field is advancing at a rapid pace, driven by continuous discoveries. For example, by 2022, more than 5600 clinical trials had been launched to evaluate anti-PD-1 or anti-PD-L1 antibodies [3]. With strong engagement from the scientific community, pharmaceutical industry, and governments worldwide, we can expect future breakthroughs in immunotherapeutic agents and novel treatment regimens with improved efficacy and safety. In this Special Issue, “Tumor Immunopharmacology”, we present contributions that highlight diverse aspects of this evolving discipline.

Safety remains a critical attribute of any therapy. For immunotherapeutics, the monitoring of adverse effects (AEs) is particularly important because (i) rare AEs may not emerge during clinical trials given the limited number of patients; and (ii) immune-related AEs can arise in off-target organs due to the systemic administration of these drugs. Thus, real-world clinical practice continually refines our understanding of the AE profiles of immunotherapies. In this Special Issue, Li and collaborators [4] report a case of grade 3 immune-related colitis in a patient with endometrial cancer receiving anti-PD-1 therapy. The AE required treatment discontinuation and was successfully managed with corticosteroids. This case illustrates the importance of systematic AE assessment using clinical scales and laboratory analyses. Furthermore, the authors discuss the mechanisms, risk factors, and management strategies for immune-related colitis.

Another critical challenge is the identification of biomarkers that can stratify patients by their likelihood of responding to current immunotherapies. Since immune checkpoint inhibitors (ICIs) have shown no superiority over chemotherapy in advanced epithelial ovarian cancer (EOC), Lai and colleagues [5] aim to identify predictors of response. By analyzing peripheral blood mononuclear cells (PBMCs) from 69 EOC patients, they found that PD-1+/CD4+ and PD-1+/CD8+ T-cell subsets correlated with clinical outcome. These subsets (i) co-express HVEM; (ii) can be driven toward effector phenotypes; and (iii) display significantly increased tumor-killing activity when exposed to ICIs in vitro. These findings provide further rationale for ongoing ICI trials in EOC and highlight the potential of PBMC profiling to refine patient selection.

The tumor microenvironment (TME) has long been recognized as a key determinant of cancer progression and therapeutic response [6]. Two contributions in this Special Issue address its role in immunotherapy. Sabit and colleagues [7] review recent advances in single-cell molecular profiling technologies, such as scRNA-seq, for dissecting the role of non-cancer cells within tumors. These approaches have uncovered stromal cell heterogeneity and allowed for the identification of their contributions to immune evasion, therapy response, and metabolic reprogramming. The authors discuss how these technologies are likely to drive biomarker discovery and novel therapeutic targets, enabling more personalized cancer treatment. In another article, Hamza and Mohammad [8] examine the limited efficacy of immunotherapies in bone metastases. They describe the complex interplay of immune and tumor cells in this setting, emphasizing the immunosuppressive soluble and cellular signals that hinder response. They highlight both the opportunities and risks of combining immunotherapies with other treatment strategies and stress the urgent need for biomarkers that can identify patients most likely to benefit.

Adoptive cell therapy represents another major advance in immunotherapy. In this approach, a patient’s immune cells are isolated, genetically modified ex vivo to enhance their anti-tumor capacity, and reinfused. In particular, chimeric antigen receptor (CAR) T-cells are engineered to recognize tumor antigens independently of MHC presentation. Hernández-López and colleagues [9] review the mechanisms of action and current clinical applications of FDA-approved CAR-T therapies in hematologic malignancies. They also discuss strategies to extend CAR-T therapy to solid tumors, as well as its potential application in chronic non-malignant diseases and infectious diseases.

The interplay between cancer cells and the immune system also provides opportunities for novel therapeutic targets. Pacheco-Hernández et al. [10] investigate the role of the transcription factor CTCF in regulating IL-6 expression in breast cancer (BrCa), as aberrant IL-6 expression is associated with metastasis and therapy resistance. The authors identify CTCF binding sites in the IL-6 promoter and show an inverse correlation between CTCF and IL-6 expression in low-tumorigenic ER+ breast cancers and in patients with good prognosis. However, in highly tumorigenic cells and aggressive breast cancers, CTCF fails to suppress IL-6 expression. Given the established roles of IL-6 in therapy resistance, stemness, and PD-1-mediated T-cell exhaustion [11], targeting the CTCF–IL-6 axis may represent a promising therapeutic strategy.

In conclusion, advances in tumor immunology, pharmacology, and translational oncology continue to drive the rapid evolution of tumor immunopharmacology. The evidence presented in this Special Issue underscores the promise of new strategies capable of delivering more effective and stable therapeutic responses for cancer patients.
